# LncRNA ZFAS1 protects chondrocytes from IL-1β-induced apoptosis and extracellular matrix degradation via regulating miR-7-5p/FLRT2 axis

**DOI:** 10.1186/s13018-023-03802-9

**Published:** 2023-04-25

**Authors:** Jicheng Han, Zongjian Luo, Yifei Wang, Yantao Liang

**Affiliations:** 1 Department of Orthopedics, Affiliated Hospital of Changchun University of Chinese Medicine, Changchun, 130021 China; 2 Department of Pathology, Jilin Cancer Hospital, Changchun, 130012 China; 3 Surgery of Bone and Soft Tissue Tumors, Jilin Cancer Hospital, 1018 Huguang Road, Chaoyang District, Changchun, 130012 China

**Keywords:** ZFAS1, miR-7-5p, FLRT2, Osteoarthritis, Chondrocytes

## Abstract

**Background:**

Increasing evidence suggested that long non-coding RNAs (lncRNAs) played vital roles in osteoarthritis (OA) progression. In this study, we aimed to reveal the protective roles of lncRNA ZFAS1 in osteoarthritis (OA) and further investigated its underlying mechanism.

**Methods:**

The chondrocytes were stimulated by IL-1β to establish an in vitro OA model. Then, the expression of ZFAS1, miR-7-5p, and FLRT2 in chondrocytes was determined by qRT-PCR. Gain- and loss-of-function assays of ZFAS1, miR-7-5p and FLRT2 were conducted. CCK-8 assay and flow cytometry analysis were performed to detect cell viability and apoptosis rate. The expression levels of cartilage-related proteins, including MMP13, ADAMTS5, Collagen II, and Aggrecan, were measured by western blot analysis. The interaction between ZFAS1 and miR-7-5p, as well as miR-7-5p and FLRT2, was confirmed by dual-luciferase reporter assay and RNA immunoprecipitation assay.

**Results:**

The expression of ZFAS1 and FLRT2 was down-regulated, while the expression of miR-7-5p was up-regulated in chondrocytes exposed to IL-1β. ZFAS1 overexpression promoted cell viability and suppressed apoptosis in IL-1β-treated chondrocytes. Besides, ZFAS1 overexpression suppressed the expression of MMP13 and ADAMTS5, but promoted the expression of Collagen II and Aggrecan to suppress ECM degradation. The mechanistic study showed that ZFAS1 sponged miR-7-5p to regulate FLRT2 expression. Furthermore, the overexpression of miR-7-5p could neutralize the effect of ZFAS1 in IL-1β-treated chondrocytes, and suppression of FLRT2 counteracted the miR-7-5p down-regulation role in IL-1β-treated chondrocytes.

**Conclusions:**

ZFAS1 could promote cell viability of IL-1β-treated chondrocytes via regulating miR-7-5p/FLRT2 axis.

*Trial registration* Not applicable.

**Supplementary Information:**

The online version contains supplementary material available at 10.1186/s13018-023-03802-9.

## Background

Osteoarthritis (OA), a chronic and degenerative joint disease, is the most common form of arthritis and a leading cause of deformity and disability in elderly individuals [1]. OA is characterized by articular cartilage degradation, limited synovitis inflammation, osteophyte formation, and subchondral bone [2, 3]. Several risk factors for OA have been identified, including age, sex, obesity, genetics, prior joint injury, and abnormal joint shape [4, 5]. Although the etiology of OA is multifactorial, there is no therapeutic method available yet to modify OA progression. Some evidence has suggested that the synthesis and degradation of cartilage might be associated with the abnormal expression of specific genes in chondrocytes, which are the only cell type that dominates the degenerative process of mature cartilage [6, 7]. Therefore, investigations of new functional genes and molecular mechanisms in OA are of great importance.

So far, several epigenetic regulations have been investigated in OA pathogenesis, including DNA methylation, histone modifications, and non-coding RNAs [8]. Particularly, recent evidences showed that non-coding RNAs played essential roles in cartilage development and OA [9–12]. Long non-coding RNAs (lncRNAs) are a class of non-coding RNAs with a length of more than 200 nucleotides; they have been reported to be the main regulators in the metastasis of tumors, the apoptosis of chondrocytes, and the metabolism of cartilage matrix [13, 14]. LncRNA ZFAS1 is reported to be an oncogene in hepatocellular carcinoma, gastric cancer, osteosarcoma, and glioma [15–18]. In studies of OA, the expression of ZFAS1 is down-regulated in OA chondrocytes compared with normal chondrocytes [19, 20]. And overexpression of ZFAS1 promotes the OA chondrocytes viability, proliferation, and migration [19]. In addition, ZAFS1 has been shown to serve as a competitive endogenous RNA (ceRNA) in several human cancers, which sponges miRNAs to regulate tumor progression indirectly [21]. In OA, Li et al*.* reported that ZFAS1 suppresses chondrocytes apoptosis through regulating miR-302d-3p/SMAD2 [20]. Nevertheless, the role and mechanism of ZFAS1 in OA remain largely unknown. miRNAs are a type of small non-coding RNAs in a length of 18–22 nucleotides. The abnormal expression of miRNA has been found to be associated with the occurrence and development of OA [22]. For example, miR-455 overexpression protects cartilage degeneration in a mouse OA model [23]. Zhang et al*.* found that the miR-132 expression is decreased in OA patients, and miR-132 overexpression elevates cell proliferation and decreased apoptosis of chondrocytes [24]. Here, we identified miR-7-5p as the downstream target of ZFAS1 in OA. miR-7-5p is the most investigated miRNA sequence in the miR-7 family. Previous studies have revealed the anti/pro-tumor effect of miR-7-5p in different human cancers [25, 26]. Another study by Huang et al*.* found the down-regulated expression of miR-7-5p in OA samples via bioinformatic analysis [27]. However, the involvement of miR-7-5p in OA metastasis and the underlying regulatory between ZFAS1 and miR-7-5p remains to be elucidated.

FLRT2 is a fibronectin leucine-rich transmembrane protein family member, which encodes a small proteoglycan located in the extracellular matrix [28]. Moreover, FLRT2 has been reported to function in receptor signaling and cell adhesion, and its overexpression in chondrogenic cells has been found to alter ERK phosphorylation levels [29]. In this study, we investigated FLRT2, which has not been studied in OA, as a downstream regulator in the ZFAS1-related ceRNA network of OA progression.

Interleukin-1β (IL-1β) is a primary inflammatory factor that plays a central role in several pathological features of OA [30]. In addition, the detrimental effects of IL-1β have been reported on the integrity of extracellular matrix (ECM) and chondrocyte function [30, 31]. In this study, OA chondrocytes induced by IL-1β were used as an in vitro model. We explored the role of ZFAS1 in promoting chondrocytes proliferation, inhibiting chondrocytes apoptosis and ECM degradation, and the underlying mechanism of regulating the miR-7-5p/FLRT2 axis. Our findings helped to clarify the mechanism of OA progression and provided a novel sight into OA treatment.

## Methods

### Cell lines and culture conditions

Human chondrocyte cell line CHON-001 was purchased from Genetimes ExCell Technology, Inc. (Shanghai, China). Cells were cultured in Dulbecco’s modified Eagle’s medium (DMEM; Gibco, NY, USA) supplemented with 10% fetal bovine serum (FBS; Gibco), 100 U/mL penicillin, and 100 μg/mL streptomycin (Gibco). Cells were incubated at 37℃ in a humidified atmosphere with 5% CO_2_.

### Chondrocyte treatment

To establish the OA model in vitro, chondrocytes were stimulated with IL-1β (R&D Systems, Minneapolis, MN, USA). In brief, when the cells reached a confluence of 70%, the cell culture medium was changed with the medium containing different quantities of IL-1β (0, 1, 5, 10, or 15 ng/mL) for 24 h, and the medium containing 10 ng/mL of IL-1β for different time (0, 6, 12, 24, and 48 h). In the functional studies, the chondrocytes were pre-exposed to ZFAS1 overexpression vector, short hairpin RNA (shRNA) targeting ZFAS1 (sh-ZFAS1), miR-7-5p mimics, miR-7-5p inhibitor, small interfering RNA (siRNA) targeting FLRT2 (si-FLRT2), and their negative controls followed IL-1β (10 ng/mL) for 24 h. All the oligonucleotides or vectors were bought from GenePharma (Shanghai, China), and the transfection was performed with Lipofectamine 2000 (Invitrogen, Carlsbad, CA, USA). After 48 h transfection, the cells were collected and then used for further experiments. The oligonucleotides are listed in Additional file 1: Table S1.

### Quantitative real-time PCR (qRT-PCR)

Total RNAs were isolated from chondrocytes by using TRIzol reagent (Invitrogen, USA) following the manufacturer’s protocol. Then, the RNA was reverse-transcribed into cDNA by using the HiScript II One-Step RT-PCR Kit (Vazyme, Nanjing, China) or miScript Reverse Transcription Kit (Qiagen, Frankfurt, Germany). qRT-PCR was performed on a 7500 Real-Time PCR system (Applied Biosystem) with SYBR Premix Dimer Eraser Kit (Takara, Japan). U6 and GAPDH were used as endogenous controls. The relative quantification was determined by the 2^−ΔΔCt^ method. All used primer sequences are listed in Additional file 1: Table S1.

### Cell viability assay

Cell Counting Kit-8 (CCK-8; Dojindo) was applied in this assay to analyze the viability of chondrocytes according to the manufacturer’s protocol. In brief, cells were inoculated into 96-well plates (2000 cells/well) and incubated for 0, 24, 48, 72, and 96 h, respectively. Then, 10 μL of CCK-8 reagent was added into the cell culture and incubated for another 2 h at 37 °C. The OD_450nm_ value was measured by a Microplate Reader (Bio-Rad Laboratories).

### Flow cytometry

The cell apoptosis rate was measured by using the Annexin V-FITC/PI Apoptosis Detection Kit (Sigma). In brief, cells were collected and washed twice with PBS, resuspended in binding buffer, and then stained with Annexin V-FITC and PI staining solutions for 30 min at room temperature. A FACSCalibur flow cytometer (BD Bioscience) was applied to analyze the apoptosis rate. Apoptosis rate results were judged as follows: Annexin V-FITC on the horizontal axis and PI on the vertical axis. The left upper quadrant was the necrotic cells; the right upper quadrant was the late apoptotic cells; the left lower quadrant was the normal cells; the right lower quadrant was the early apoptotic cells. Apoptosis rate was defined as both early apoptotic cells in the right lower quadrant and late apoptotic cells in the right upper quadrant.

### Western blot analysis

Total proteins from chondrocytes were extracted by using radioimmunoprecipitation assay lysis (RIPA) and then quantified by Pierce BCA Protein Assay Kit (Thermo Fisher Scientific). The protein extracts were separated by 10% SDS-PAGE and then transferred onto a PVDF membrane (Bio-Rad). After that, 5% skim milk was used to block the membrane for 30 min at room temperature. Next, the blots were incubated with primary antibodies obtained from Abcam: anti-FLRT2 (1/500, ab154023), anti-matrix metalloproteinase 13 (MMP13) (1/5000, ab39012), anti-ADAMTS5 (1/250, ab41037), anti-Collagen II (1/1000, ab188570), anti-Aggrecan (1 μg/mL, ab3778), and anti-β-actin (1/2000, ab8227), overnight at 4℃. Membranes were then incubated with the secondary antibody (ab7090) for 1 h at room temperature. The protein bands were measured using the enhanced chemiluminescence system (Millipore).

### Dual-luciferase reporter assay

The wild type and mutant sequences of ZFAS1 and FLRT2 containing the binding site of miR-7-5p were designed by Sangon Biotech (Shanghai, China) and cloned into pmirGLO luciferase reporter vector (Promega, USA). The reporter vector was co-transfected with miR-7-5p mimics or the negative control into chondrocytes by Lipofectamine 2000 (Invitrogen), respectively. After 48 h of transfection, the relative luciferase activity of the chondrocytes from each group was determined by the Dual-Luciferase Reporter Assay System (Promega).

### Subcellular localization

To investigate the subcellular localization of ZFAS1 in chondrocytes, a fluorescent in situ hybridization kit (RiboBio) was used following the instructions. Briefly, the probe targeting ZFAS1 was obtained from GenePharma (Shanghai, China). Then the anti-ZFAS1 probe was labeled with Alexa Fluor 594 and incubated with CHON-001 cells at 55 °C for 18 h. Cell nuclei were visualized by staining with 4ʹ,6-diamidino-2-phenylindole dihydrochloride (DAPI) for 30 min. The subcellular localization of ZFAS1 could be observed under a fluorescence microscope (Nikon, Japan).

### Isolation of cytoplasmic and nuclear RNA

Cytoplasmic and nuclear RNA was isolated and purified by the Cytoplasmic & Nuclear RNA Purification Kit (Norgen) according to the manufacturer’s protocol. U6 and GAPDH were taken as the internal references.

### RNA immunoprecipitation assay (RIP)

The EZ Magna RNA Immunoprecipitation Kit (Millipore) was used to confirm the interaction between ZFAS1 and miR-7-5p under the manufacturer’s instructions. In brief, the normal human chondrocyte cells were lysed using RIP lysis buffer. Then, 5 μg of anti-Ago2 antibody (Abcam) or anti-IgG antibody (Abcam) was added into the cell lysate and incubated with magnetic beads overnight at 4℃ for immunoprecipitation. IgG served as a negative control. Finally, the co-precipitated RNA fraction was purified by RIP assay and detected by qRT-PCR.

### Statistical analysis

All statistical analyses were conducted using GraphPad 9.1.1. All data were presented as the mean ± standard deviation (SD). The significant difference between groups was determined by Student’s *t*-test or one-way ANOVA. *P* < 0.05 was considered statistically significant.

## Results

### ZFAS1 expression was down-regulated in IL-1β-stimulated chondrocytes

**Fig. 1:**
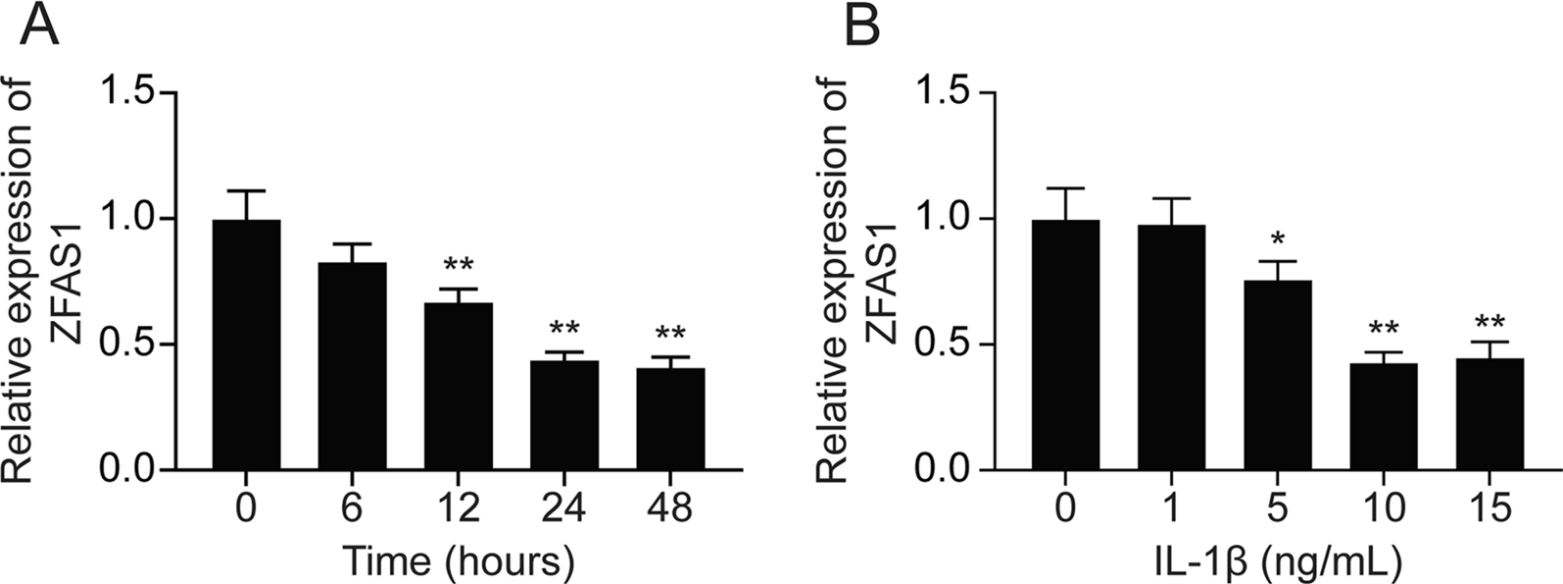
ZFAS1 was down-regulated in IL-1β-treated chondrocytes. **A** Expression level of ZFAS1 in chondrocytes treated with 10 ng/mL IL-1β for 0, 6, 12, 24, or 48 h. **P < 0.01, compared with the 0 h group. **B** Expression level of ZFAS1 in chondrocytes treated with IL-1β at different concentrations (0, 1, 5, 10, and 15 ng/mL) for 24 h. *P < 0.05, **P < 0.01, compared with 0 ng/mL group

Increasing evidence suggested that lncRNAs played vital roles in OA progression. A previous study based on microarray analysis reported four differently expressed lncRNAs (SNHG5, ZFAS1, GAS5, and DANCR) involved in OA cartilage [32]. Here, we detected the expression of the four lncRNAs in IL-1β-treated (10 ng/mL, 24 h) chondrocytes. As shown in Additional file 2: Fig. S1, the expression of GAS5 and DANCR was significantly up-regulated after IL-1β stimulation (*P* < 0.01), while the expression of SNHG5 and ZFAS1 was significantly down-regulated after IL-1β stimulation (*P* < 0.01). In the two significant down-regulated lncRNAs, ZFAS1 showed a higher fold change; thus, we selected ZFAS1 in our subsequent studies. We detected the ZFAS1 expression in chondrocytes stimulated with IL-1β at different times or with different IL-1β concentrations. qRT-PCR results showed that ZFAS1 expression was down-regulated with the time increases (Fig. 1A,* P* < 0.01). Moreover, with the concentration increasing, the ZFAS1 expression was gradually down-regulated (Fig. 1B, * P* < 0.05). Overall, these results suggested that ZFAS1 was down-regulated in the in vitro OA model.

### ZFAS1 suppressed IL-1β-induced ECM degradation and apoptosis of chondrocytes and promoted cell proliferation

**Fig. 2:**
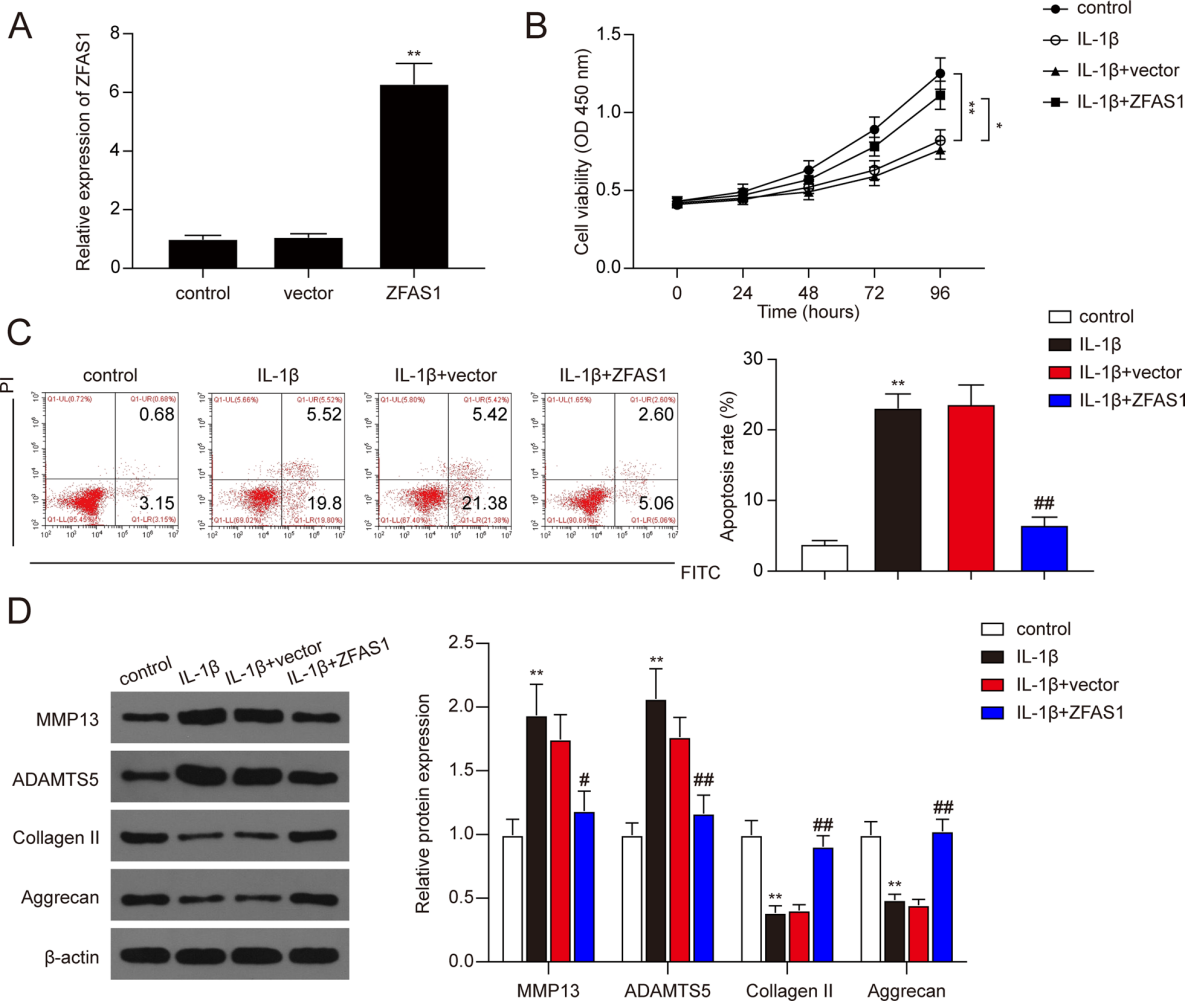
Overexpression of ZFAS1 ameliorated the apoptosis and ECM degradation in IL-1β-treated chondrocytes. **A** The expression of ZFAS1 was detected in chondrocytes treated with empty vector or ZFAS1 overexpression vector or without any treatment. **P < 0.01 compared with the vector group. **B** The effects of ZFAS1 overexpression on the cell viability of IL-1β-treated chondrocytes. *P < 0.05, **P < 0.01. **C** The effects of ZFAS1 overexpression on the apoptosis rate of IL-1β-treated chondrocytes. **P < 0.01, compared with control group; ^##^*P* < 0.01, compared with IL-1β group. **D** Relative expression levels of MMP13, ADAMTS5, Collagen II, and Aggrecan measured by western blot analysis. **P < 0.01, compared with control group; ^#^*P* < 0.05, ^##^*P* < 0.01, compared with IL-1β group

**Fig. 3:**
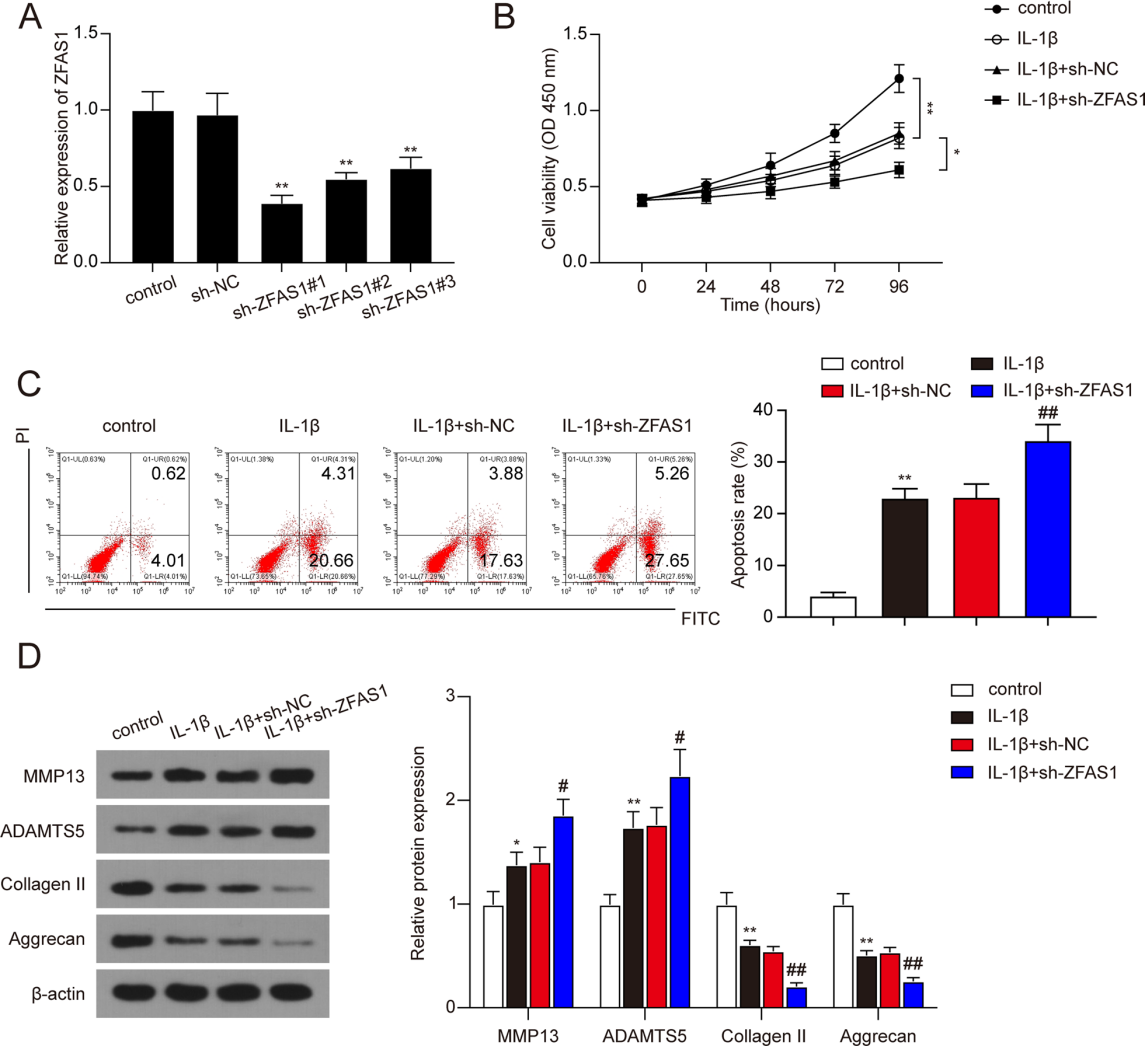
Knockdown of ZFAS1 promoted the apoptosis and ECM degradation in IL-1β-treated chondrocytes. **A** The expression of ZFAS1 was detected in chondrocytes treated with sh-NC or knockdown of ZFAS1 by sh-ZFAS1#1-3 or without any treatment. **P < 0.01 compared with the sh-NC group. **B** The effects of knockdown of ZFAS1 on the cell viability of IL-1β-treated chondrocytes. *P < 0.05, **P < 0.01. **C** The effects of knockdown of ZFAS1 on the apoptosis rate of IL-1β-treated chondrocytes. **P < 0.01, compared with control group; ^##^*P* < 0.01, compared with IL-1β group. **D** Relative expression levels of MMP13, ADAMTS5, Collagen II, and Aggrecan measured by western blot analysis. *P < 0.05, **P < 0.01, compared with control group; ^#^*P* < 0.05, ^##^*P* < 0.01, compared with IL-1β group

We then investigated the effects of ZFAS1 on cell viability and apoptosis of IL-1β-stimulated chondrocytes. The cells were transfected with ZFAS1 overexpression vector and conducted to the CCK-8 assay and flow cytometry apoptosis analysis. As shown in Fig. 2A, transfection with ZFAS1 overexpression vector significantly increased the ZFAS1 expression in chondrocytes (*P* < 0.01). Then, CCK-8 assay results showed that the cell viability of IL-1β-stimulated chondrocytes was remarkably lower than the control group (Fig. 2B, * P* < 0.01). Moreover, ZFAS1 overexpression in IL-1β-stimulated chondrocytes significantly enhanced the cell viability rate compared to the IL-1β + vector group (Fig. 2B, * P* < 0.05). In addition, flow cytometry results indicated that apoptosis rate was significantly increased after IL-1β treatment, while the ZFAS1 overexpression significantly reduced the cell apoptosis rate (Fig. 2C, * P* < 0.01). As MMP13 and ADAMTS5 were positively correlated with ECM degradation, and Collagen II and Aggrecan were negatively correlated with ECM degradation in chondrocytes. We then detect the expression of MMP13, ADAMTS5, Collagen II, and Aggrecan with western blot assay. As expected, the expression of MMP13 and ADAMTS5 were significantly up-regulated after IL-1β stimulation, while the expression of Collagen II and Aggrecan was significantly decreased after IL-1β stimulation (Fig. 2D, * P* < 0.01). And the expression changes in response to IL-1β stimulation were abolished with ZFAS1 overexpression (Fig. 2D, * P* < 0.05). In addition, knockdown of ZFAS1 was conducted for further verification. As shown in Fig. 3A–D, knockdown of ZFAS1 significantly reduced the cell viability rate while increasing cell apoptosis rate after IL-1β treatment (*P* < 0.05). In short, these results demonstrated that overexpression of ZFAS1 could promote cell viability and repress cell apoptosis and ECM degradation in IL-1β treated chondrocytes.

### ZFAS1 directly targeted miR-7-5p in chondrocytes

**Fig. 4:**
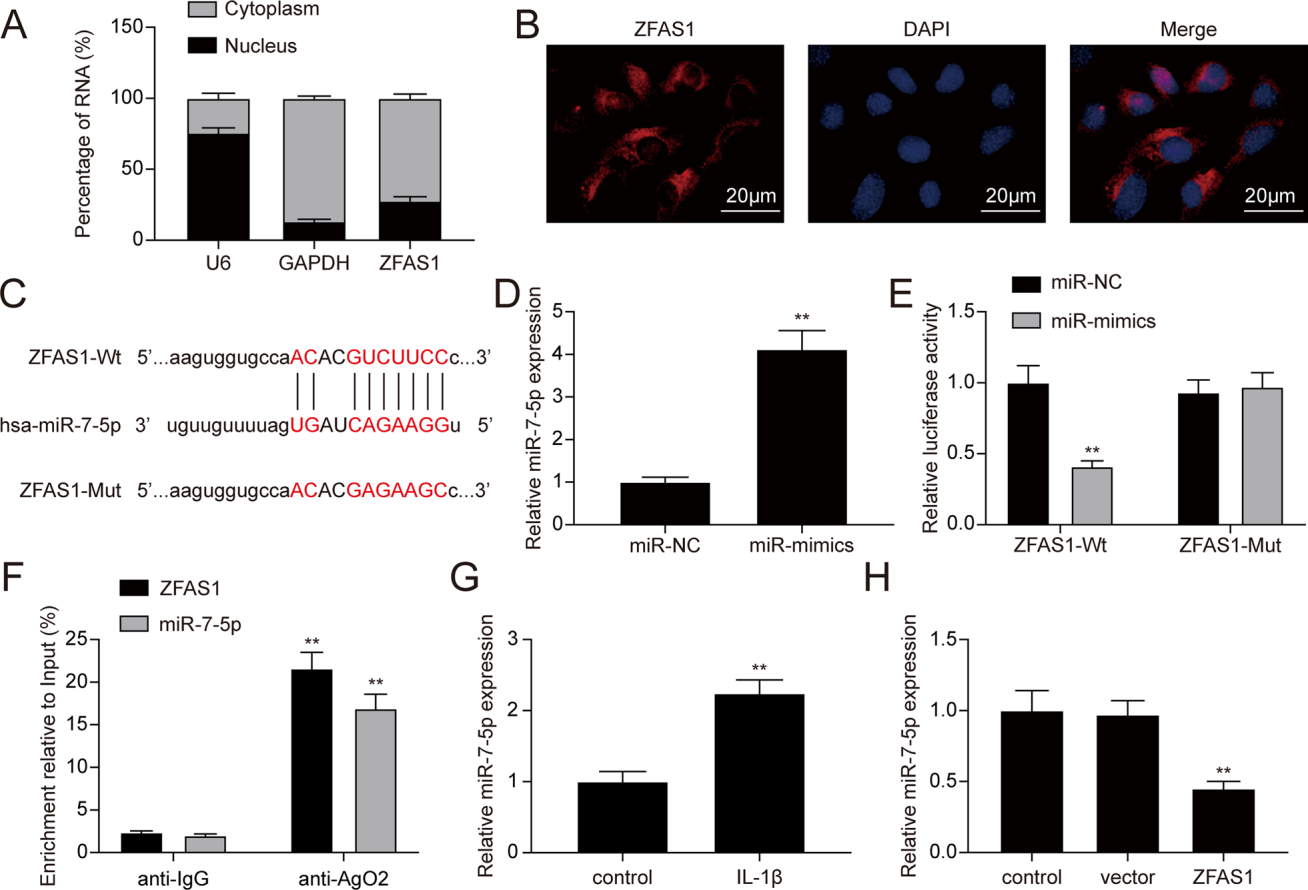
ZFAS1 directedly targeted miR-7-5p. **A** Subcellular ZFAS1 distribution was analyzed by isolating nuclear and cytoplasmic RNA fractions from chondrocytes, U6 and GAPDH were used as controls. **B** RNA-FISH was performed to verify the localization of ZFAS1 in the cytoplasm. **C** Predicted ZFAS1 binding sites with miR-7-5p. **D** Determination of miR-7-5p expression in chondrocytes transfected with miR-NC or miR-mimics. **P < 0.01, compared with the miR-NC group. **E** Dual-luciferase reported assay was applied to verify the targeting relationship between ZFAS1 and miR-7-5p. **P < 0.01, compared with the miR-NC group. **F** RIP with anti-Ago2 and anti-IgG antibodies were performed to analyze endogenous Ago2 binding to RNA. **P < 0.01, compared with the anti-IgG group. **G** Detection of miR-7-5p expression in chondrocytes with or without IL-1β treatment. **P < 0.01, compared with the control group. **H** Overexpression of ZFAS1 could suppress miR-7-5p expression in chondrocytes. **P < 0.01, compared with the vector group

lncRNAs have been reported to sponge miRNAs and serve as ceRNAs [21]. Hence, we detected the expression distribution of ZFAS1 in chondrocytes. The results showed that ZFAS1 was mainly located in the cytoplasm of chondrocytes (Fig. 4A). FISH assay further confirmed the cytoplasm location of ZFAS1 in chondrocytes (Fig. 4B). With the starBase webtool, we found that ZFAS1 had binding sites to miR-7-5p (Fig. 4C). The dual-luciferase reporter assay was performed to confirm the targeting relationship. Transfection of miR-7-5p mimics significantly promoted the miR-7-5p expression in chondrocytes (Fig. 4D, * P* < 0.01). Then, the luciferase activity of the ZFAS1-Wt luciferase reporter vector was decreased with miR-7-5p overexpression, while the luciferase activity in the ZFAS1-Mut luciferase vector had no obvious changes with miR-7-5p overexpression (Fig. 4E, * P* < 0.01). For further confirmation, we performed the RIP assay, and the results showed that both ZFAS1 and miR-7-5p were enriched in Ago2 immunoprecipitation compared to IgG immunoprecipitation (Fig. 4F, * P* < 0.01), indicating that ZFAS1 and miR-7-5p had a targeting relationship in chondrocytes. Moreover, the qRT-PCR results manifested that miR-7-5p expression was remarkably increased in IL-1β-treated chondrocytes (Fig. 4G, * P* < 0.01). Additionally, the overexpression of ZFAS1 could down-regulate the expression of miR-7-5p in chondrocytes (Fig. 4H, * P* < 0.01). The above results demonstrated that ZFAS1 could sponge miR-7-5p and suppress its expression in chondrocytes.

### miR-7-5p could neutralize the effects of ZFAS1 overexpression on viability, apoptosis, and ECM degradation in IL-1β-stimulated chondrocytes

**Fig. 5:**
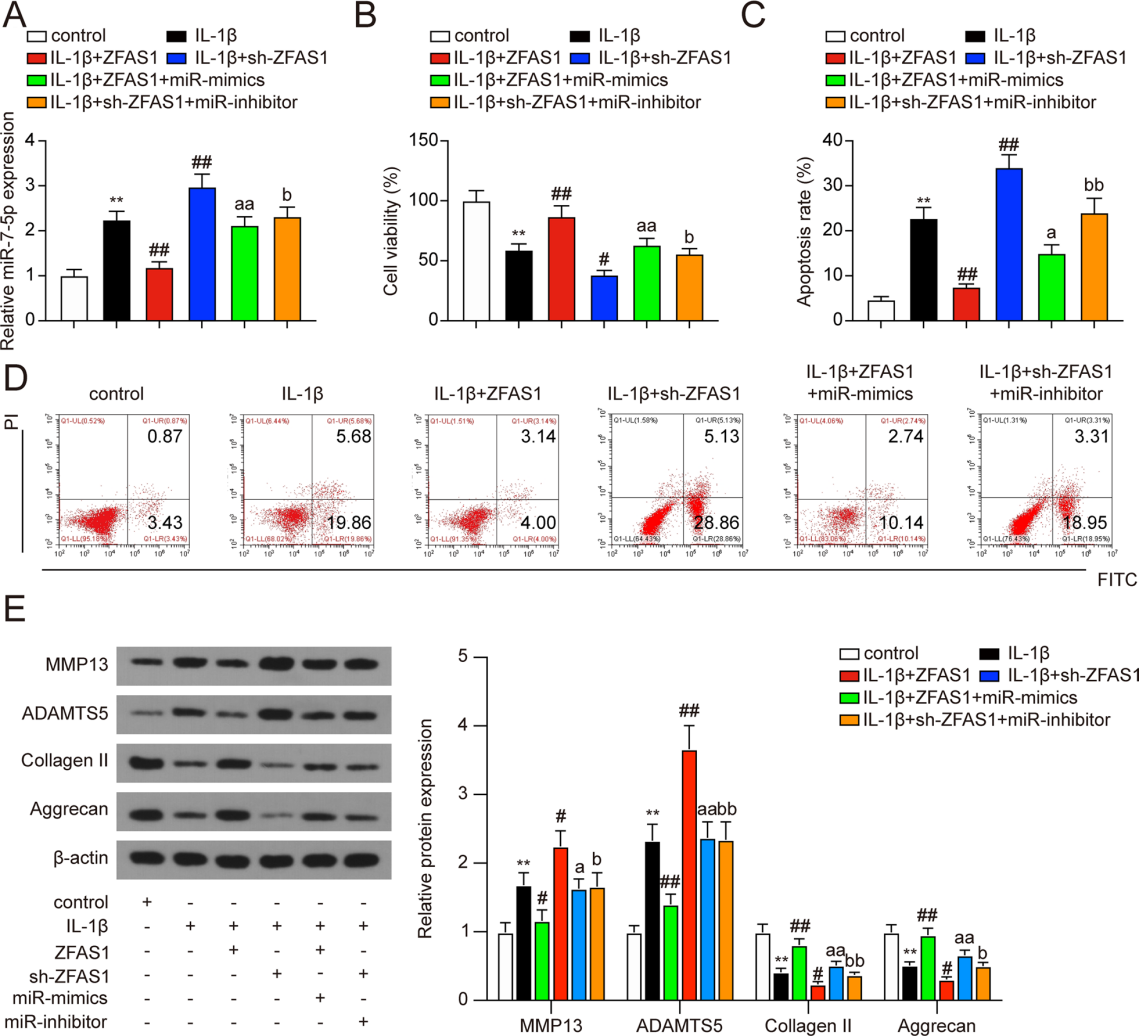
miR-7-5p could neutralize the effects of ZFAS1 overexpression on IL-1β-treated chondrocytes. **A** qRT-PCR detection of miR-7-5p expression. **P < 0.01, compared with the control group; ^##^*P* < 0.01, compared with IL-1β group; ^aa^*P* < 0.01, compared with IL-1β + ZFAS1 group; ^b^*P* < 0.05, compared with IL-1β + sh-ZFAS1 group. **B** Effects of ZFAS1 and miR-7-5p on the cell viability of IL-1β-treated chondrocytes. **P < 0.01, compared with the control group; ^#^*P* < 0.05, ^##^*P* < 0.01, compared with IL-1β group; ^aa^*P* < 0.01, compared with IL-1β + ZFAS1 group; ^b^*P* < 0.05, compared with IL-1β + sh-ZFAS1 group. **C**–**D** Effect of ZFAS1 and miR-7-5p on the cell apoptosis of IL-1β-treated chondrocytes. **P < 0.01, compared with the control group; ^##^*P* < 0.01, compared with IL-1β group; ^a^*P* < 0.05, compared with IL-1β + ZFAS1 group; ^bb^*P* < 0.01, compared with IL-1β + sh-ZFAS1 group. **D** Relative expression levels of MMP13, ADAMTS5, Collagen II, and Aggrecan after cell transfection were detected by western blot. **P < 0.01, compared with the control group; ^#^*P* < 0.05, ^##^*P* < 0.01, compared with IL-1β group; ^a^*P* < 0.05, ^aa^*P* < 0.01, compared with IL-1β + ZFAS1 group; ^b^*P* < 0.05, ^bb^*P* < 0.01, compared with IL-1β + sh-ZFAS1 group

In order to confirm the regulatory function of the ZFAS1/miR-7-5p axis in IL-1β-stimulated chondrocytes, rescue experiments were performed. As shown in Fig. 5A, miR-7-5p expression was suppressed in chondrocytes with ZFAS1 overexpression (*P* < 0.01), and this down-regulation was then compensated by increasing miR-7-5p expression via miR-7-5p mimics co-transfection (*P* < 0.01). Subsequently, the results of CCK-8 assay and flow cytometry analysis indicated that the promoting effect of ZFAS1 overexpression on cell viability and its inhibitory effect on cell apoptosis could be partly neutralized by co-transfection with miR-7-5p mimics (Fig. 5B–D, * P* < 0.05). We next analyzed the effects of miR-7-5p on the ZFAS1-mediated modulation of the ECM in chondrocytes. The results showed that ZFAS1 overexpression led to MMP13 and ADAMTS5 down-regulation and Collagen II and Aggrecan up-regulation, which was overall blocked by increasing miR-7-5p expression in chondrocytes (Fig. 5E, * P* < 0.05). Rescue experiments were also performed with sh-ZFAS1 and miR-7-5p inhibitor, and the results indicated that the effects of ZFAS1 knockdown on IL-1β-treated chondrocytes could be partly neutralized by co-transfection with miR-7-5p inhibitor (Fig. 5A–E, * P* < 0.05). To conclude, these results suggested a ZFAS1/miR-7-5p interaction in regulating OA chondrocytes viability, apoptosis, and ECM degradation.

### miR-7-5p directly targeted FLRT2

**Fig. 6:**
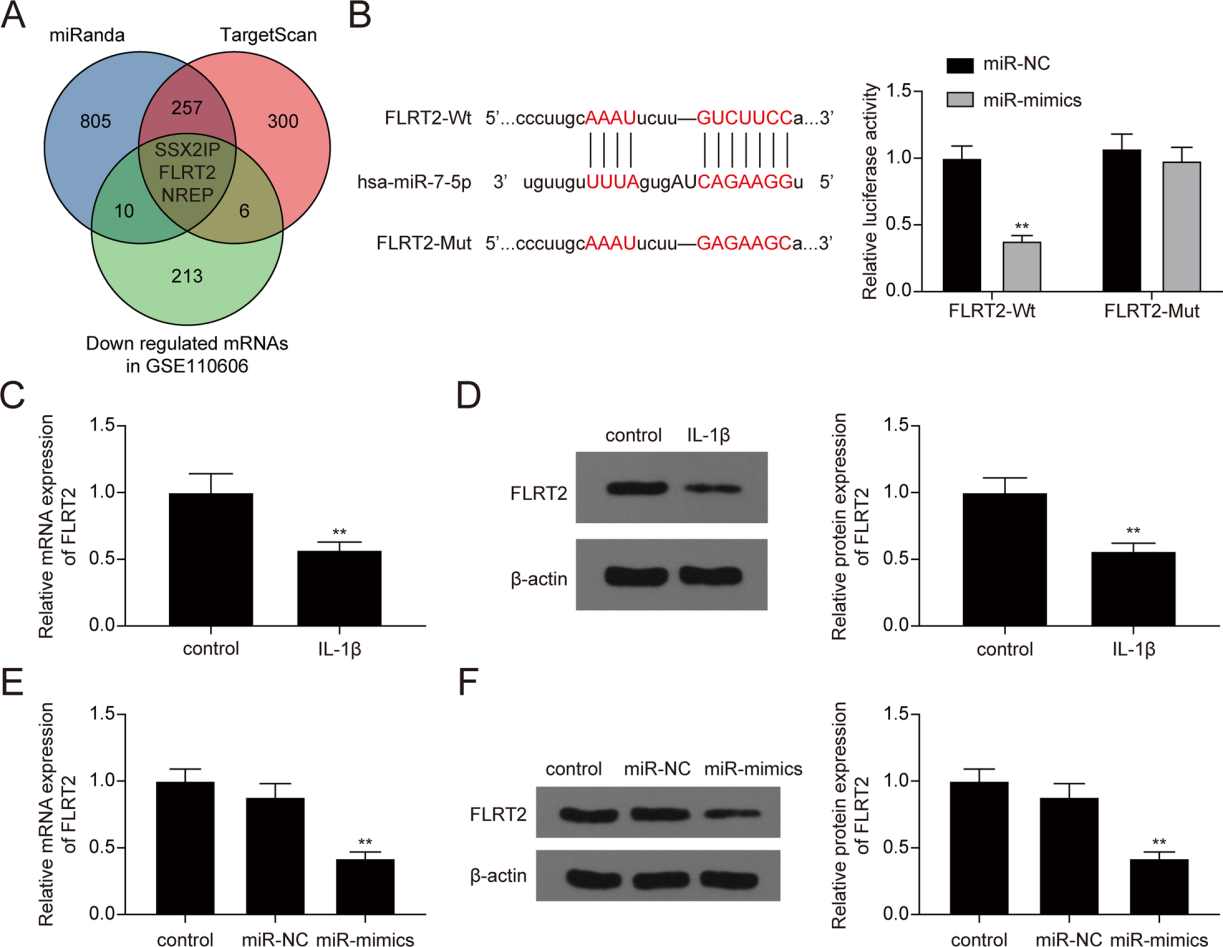
FLRT2 was a target of miR-7-5p. **A** Three mRNAs including SSX2IP, FLRT2, and NREP were screened out by performing interaction analysis. **B** Dual-luciferase reported assay was applied to verify the targeting relationship between miR-7-5p and FLRT2. **P < 0.01, compared with the miR-NC group. **C**–**D** FLRT2 mRNA **C** and protein **D** expressions were significantly decreased in IL-1β-treated chondrocytes. **P < 0.01, compared with the control group. **E**–**F** FLRT2 mRNA and protein expressions were significantly decreased following the transfection with miR-7-5p mimics. **P < 0.01, compared with the miR-NC group

**Fig. 7:**
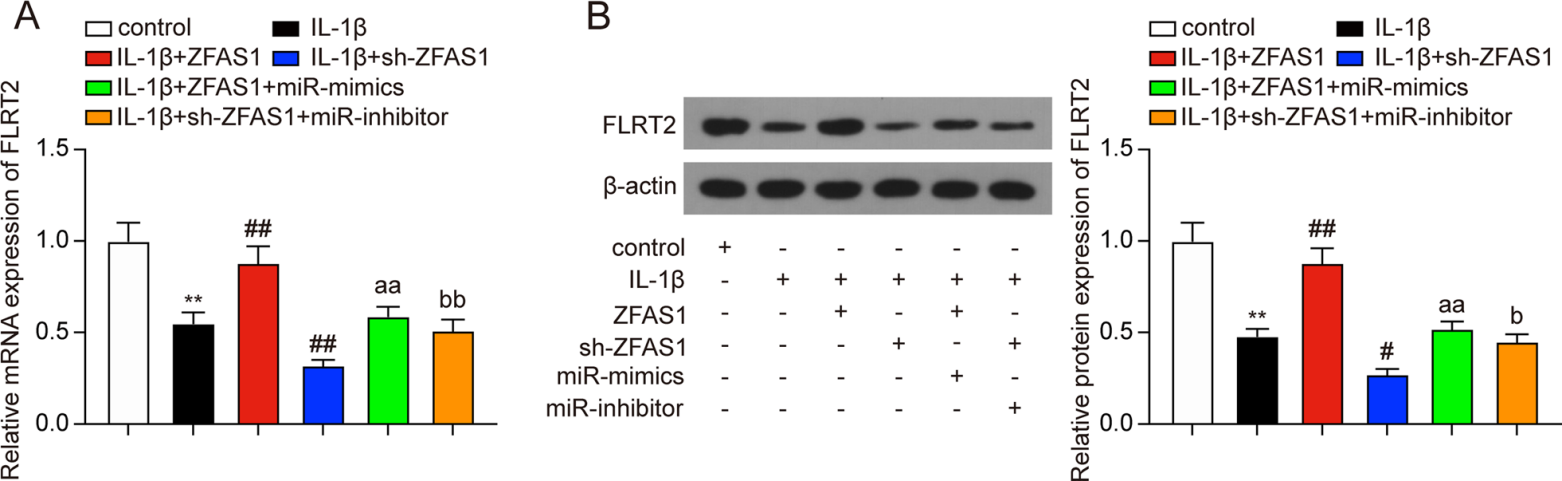
FLRT2 expression was regulated by ZFAS1/miR-7-5p in IL-1β-treated chondrocytes. (A-B) FLRT2 mRNA **A** and protein **B** expressions after transfection. **P < 0.01, compared with the control group; ^#^*P* < 0.05, ^##^*P* < 0.01, compared with IL-1β group; ^aa^*P* < 0.01, compared with IL-1β + ZFAS1 group; ^b^*P* < 0.05, ^bb^*P* < 0.01, compared with IL-1β + sh-ZFAS1 group

miRNA could bind to the 3'UTR of target mRNAs and subsequently induce their degradation [33]. In the current study, we used miRanda and TargetScan to predict the targets of miR-7-5p and interacted the results with the down-regulated mRNAs in GSE110606 (Fig. 6A, Additional file 3: Table S2). Among the three candidate mRNAs (SSX2IP, FLRT2, and NREP), FLRT2 was previously reported to promote cellular proliferation and inhibit cell adhesion during chondrogenesis [34]. Thus, we selected FLRT2 as the potential targeted gene of miR-7-5p. The binding sites between miR-7-5p and FLRT2 are shown in Fig. 6B. Dual-luciferase reporter assay confirmed the targeting relationship that miR-7-5p mimics significantly decreased the luciferase activity of FLRT2-Wt reporter, but showed no significant effects on the luciferase activity of FLRT2-Mut reporter (Fig. 6B). In addition, FLRT2 mRNA and protein expression levels decreased remarkably in IL-1β-treated chondrocytes (Fig. 6C, D, * P* < 0.01). Furthermore, qRT-PCR and western blot results confirmed that miR-7-5p overexpression could remarkably suppress FLRT2 expression in chondrocytes (Fig. 6E, F, * P* < 0.01). Besides, ZFAS1 overexpression led to high expression of FLRT2 IL-1β-treated chondrocytes, and this was rescued by co-transfection with miR-7-5p mimics (Fig. 7A, B, * P* < 0.01). In addition, knockdown of ZFAS1 resulted in down-regulation of FLRT2 in IL-1β-treated chondrocytes, and this was rescued by co-transfection with miR-7-5p inhibitors (Fig. 7A, B, * P* < 0.05). Thus, it was indicated that FLRT2 was downstream of ZFAS1/miR-7-5p in chondrocytes.

### FLRT2 suppression could neutralize the effects of miR-7-5p inhibition on IL-1β-treated chondrocytes

**Fig. 8:**
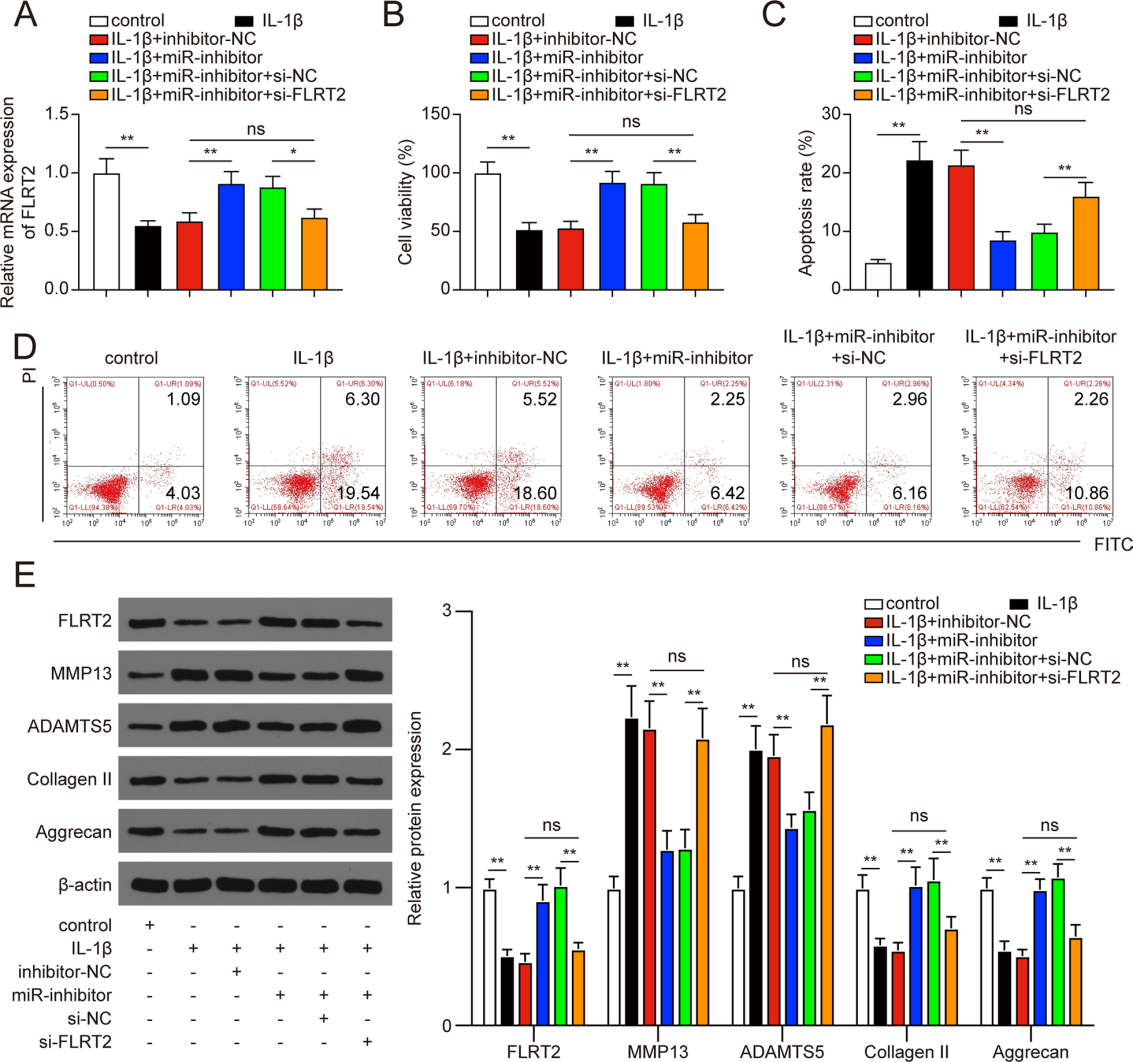
FLRT2 could counteract the effects of miR-7-5p inhibition on IL-1β-treated chondrocytes. **A** FLRT2 expression was promoted in chondrocytes transfected with miR-7-5p inhibitor and restored in chondrocytes co-transfected with miR-7-5p inhibitor and si-FLRT2. *P < 0.05, **P < 0.01; ns, not significant. **B**–**D** The suppression of FLRT2 could neutralize the effects of miR-7-5p inhibition on the viability (**B**) and apoptosis (**C**–**D**) of IL-1β-treated chondrocytes. **P < 0.01; ns, not significant. **E** Relative expression levels of FLRT2, MMP13, ADAMTS5, Collagen II, and Aggrecan after transfection were detected by western blot analysis. **P < 0.05; ns, not significant

Since the targeting relationship between FLRT2 and miR-7-5p was confirmed, we next wanted to explore whether the effects of miR-7-5p inhibition on the cell viability, apoptosis, and ECM degradation could be restored by FLRT2 suppression in IL-1β-treated chondrocytes. miR-7-5p inhibition markedly promoted FLRT2 expression in chondrocytes, and this effect was restored by co-transfection with FLRT2 siRNA (Fig. 8A, * P* < 0.05). In CCK-8 assay, we found that the inhibition of miR-7-5p could markedly increase the viability of IL-1β-treated chondrocytes (Fig. 8B, * P* < 0.01). However, the effect of miR-7-5p inhibition was diminished after the co-transfection with FLRT2 siRNA (Fig. 8B, * P* < 0.01). On the other hand, the apoptosis rates of IL-1β-treated chondrocytes were significantly repressed in miR-7-5p inhibited group, while the repression was neutralized when co-transfected with FLRT2 siRNA (Fig. 8C-D, * P* < 0.01). Subsequently, western blot results showed that the expression of MMP13 and ADAMTS5 was decreased obviously with miR-7-5p inhibition, while the co-transfection of miR-7-5p inhibitor and FLRT2 siRNA could restore the MMP13 and ADAMTS5 expression (Fig. 8E, * P* < 0.01). Oppositely, the expression of Collagen II and Aggrecan was up-regulated with miR-7-5p inhibition, and this up-regulation was abrogated by co-transfection with miR-7-5p inhibitor and FLRT2 siRNA (Fig. 8E, * P* < 0.01). In brief, the above results implied a miR-7-5p/FLRT2 axis in regulating OA chondrocytes viability, apoptosis, and ECM degradation.

## Discussion

OA as a degenerative joint disease shows a high incidence in middle-aged and elderly individuals. It is characterized by the destruction of articular cartilage and can lead to joint stiffness and loss of motor ability [35]. Previous studies have reported the association between the pathogenesis and development of OA and lncRNAs. For instance, lncRNA-CIR, related to cartilage injury, is able to induce in vitro degradation of cartilage extracellular matrix [36]; lncRNA HOTAIR overexpression could contribute to IL-1β-induced chondrocyte apoptosis and matrix metalloproteinases overexpression [37]. However, the expression profile of lncRNAs and their potential targets as well as biological functions in terms of OA development remain elusive. In this study, the effect of lncRNA ZFAS1 in OA development was explored.

IL-1β was commonly used to simulate chondrocytes affected by OA. As reported, IL-1β could promote the secretion of pro-inflammatory cytokines and suppress collagen synthesis, thereby resulting in the biological dysfunction of chondrocytes and the degradation of articular cartilage [38]. Here, we found that IL-1β treatment decreased the cell viability and increased the apoptosis of chondrocytes. We also found that IL-1β treatment promoted MMP-13 and ADAMTS5 expression and suppressed Collagen II and Aggrecan expression to stimulate ECM destruction in chondrocytes, which confirmed the effect of IL-1β on aggravating OA development.

ZFAS1 has been reported as an essential player in promoting chondrocyte proliferation and suppressing matrix synthesis in OA, and it works through Wnt3a [19]. In consist with the previous study, we found that ZFAS1 expression was decreased in IL-1β-treated chondrocytes. Functionally, overexpression of ZFAS1 promoted the viability and suppressed the apoptosis of IL-1β-treated chondrocytes. Furthermore, overexpression of ZFAS1 could inhibit IL-1β-induced ECM degradation, which has not been reported before. Besides, we found that ZFAS1 is mainly located in the cytoplasm of chondrocytes, suggesting a potential of ZFAS1 as a ceRNA. Li et al*.* found ZFAS1 sponged miR-302d-3p to regulate SMAD2 expression in OA [20]. In this study, we identified miR-7-5p as a downstream target of ZFAS1, which was also reported by Peng et al*.* [39] and Mo et al*.* [40].

According to the qRT-PCR results, miR-7-5p was highly expressed in IL-1β-treated chondrocytes. Chen et al*.* reported that miR-7-5p expression is up-regulated in OA knee cartilage tissues and IL-1β-stimulated OA chondrocytes [41]. Zhou et al*.* also found an up-regulated expression of miR-7 in chondrocytes induced by IL-1β [42]. Our result was consistent with the previous studies. Furthermore, we found that the overexpression of miR-7-5p could notably reduce the function of ZFAS1 overexpression with respect to cell viability, apoptosis and ECM degradation in IL-1β-stimulated chondrocytes. All these results suggested that ZFAS1 regulated cell viability, apoptosis, and ECM degradation by functioning as a sponge for miR-7-5p in chondrocytes stimulated by IL-1β.

We then identified FLRT2 as the downstream target of miR-7-5p. FLRT2 belonged to the fibronectin leucine-rich transmembrane protein family, which are essential factors in guiding several biological processes, such as neural, vascular, and early embryonic development [43–45]. It was reported that FLRT2 played an essential role in regulating cell adhesion and cell–matrix interactions during early chondrogenesis [46, 47]. In addition, Xu et al*.* [34] identified that FLRT2 was overexpressed in ATDC5 cells and could promote cell proliferation and reduce intercellular adhesion at early chondrogenesis. In this study, dual-luciferase reporter assay confirmed the targeting relationship between miR-7-5p and FLRT2. In addition, FLRT2 suppression could neutralize the viability, apoptosis, and matrix synthesis of OA chondrocytes, which were mediated by miR-7-5p inhibition. More importantly, the FLRT2 expression was significantly increased in OA chondrocytes following ZFAS1 overexpression, indicating that FLRT2 was regulated by ZFAS1. Therefore, ZFAS1 might regulate the FLRT2 expression indirectly via sponging miR-7-5p, and we found the regulatory network of ZFAS1/miR-7-5p/FLRT2 in OA.

## Conclusions

In summary, our study demonstrated that ZFAS1 could enhance proliferation and inhibit apoptosis and matrix synthesis in IL-1β-treated chondrocytes via regulating miR-7-5p/FLRT2 axis. ZFAS1 functioned as a novel ceRNA might serve as a potential therapeutic target for OA treatment. Nevertheless, the regulatory effects of ZFAS1 on OA development still require further demonstration by in vivo studies with animal models.

## Supplementary Information

**Additional file 1: Table S1.** The sequences of oligonucleotides and primers

**Additional file 2: Figure S1.** lncRNAs expression changes in chondrocytes treated with IL-1β. **A**–**D** The expression levels of four lncRNAs were determined by qRT-PCR analysis. The chondrocytes were treated with 10 ng/mL IL-1β for 24 h. **P < 0.01, compared with the control group

**Additional file 3: Table S2.** The list of the down-regulated genes in GSE110606

## Data Availability

The datasets used and analyzed during the current study are available from the corresponding author on reasonable request.

## Abbreviations

CCK-8: Cell Counting Kit-8
ceRNA: Competitive endogenous RNA
DAPI: 4ʹ,6-Diamidino-2-phenylindole dihydrochloride
DMEM: Dulbecco’s modified Eagle’s medium
ECM: Extracellular matrix
FBS: Fetal bovine serum
lncRNAs: Long non-coding RNAs
IL-1β: Interleukin-1β
MMP13: Matrix metalloproteinase 13
OA: Osteoarthritis
RIP: RNA immunoprecipitation assay
SD: Standard deviation
shRNA: Short hairpin RNA
siRNA: Small interfering RNA
