# Supplementary material for: LncRNA ZFAS1 protects chondrocytes from IL-1β-induced apoptosis and extracellular matrix degradation via regulating miR-7-5p/FLRT2 axis
Source: J Orthop Surg Res. 2023 Apr 25;18:320. doi: 10.1186/s13018-023-03802-9 (PMC10131303; doi:10.1186/s13018-023-03802-9)
Supplement: Supplementary file 1 — Additional file 1: Table S1. The sequences of oligonucleotides and primers [file 13018_2023_3802_MOESM1_ESM.docx]

Table S1 The sequences of oligonucleotides and primers.

| Name | Sequence |
| --- | --- |
| miR-7-5p mimics | UGGAAGACUAGUGAUUUUGUUGUU |
| mimics NC | UUCUCCGAACGUGUCACGU |
| miR-7-5p inhibitor | AACAACAAAAUCACUAGUCUUCCA |
| inhibitor NC | CAGUACUUUUGUGUAGUACAA |
| sh-ZFAS1#1 | CACCGCGAAAGCCATCTTTGGTTATCGAAATAACCAAAGATGGCTTTCGC |
| sh-ZFAS1#2 | CACCGCAGGTGCGAAAGCCATCTTTCGAAAAAGATGGCTTTCGCACCTGC |
| sh-ZFAS1#3 | CACCGCGAAAGCCATCTTTGGTTATCGAAATAACCAAAGATGGCTTTCGC |
| sh-NC | CACCGCGCGATACTTCGGTATTATAATAACGAATTATTATAATACCGAAGTATCGCGC |
| si-FLRT2 | CCAAGAAUGTCAGAGUUCUCCAUUU |
| si-NC | UUCUCCGAACGUGUCACGU |
| ZFAS1 | Forward primer: GCTATTGTCCTGCCCGTTAG |
|  | Reverse primer: TCGTCAGGAGATCGAAGGTT |
| miR-7-5p | Forward primer:  ACACTCCAGCTGGGTGGAAGACTAGTGATTTT |
|  | Reverse primer: TGGTGTCGTGGAGTCG |
| FLRT2 | Forward primer: ACCCTTGGTTTTGTGACTGC |
|  | Reverse primer: AGGACCTTGGCACATGAAAC |
| GAPDH | Forward primer: CAAGGCTGAGAACGGGAAG |
|  | Reverse primer: TGAAGACGCCAGTGGACTC |
| U6 | Forward primer: CTCGCTTCGGCAGCACA |
|  | Reverse primer: AACGCTTCACGAATTTGCGT |
