# Supplementary figures and images for: LncRNA ZFAS1 protects chondrocytes from IL-1β-induced apoptosis and extracellular matrix degradation via regulating miR-7-5p/FLRT2 axis
Source: J Orthop Surg Res. 2023 Apr 25;18:320. doi: 10.1186/s13018-023-03802-9 (PMC10131303; doi:10.1186/s13018-023-03802-9)

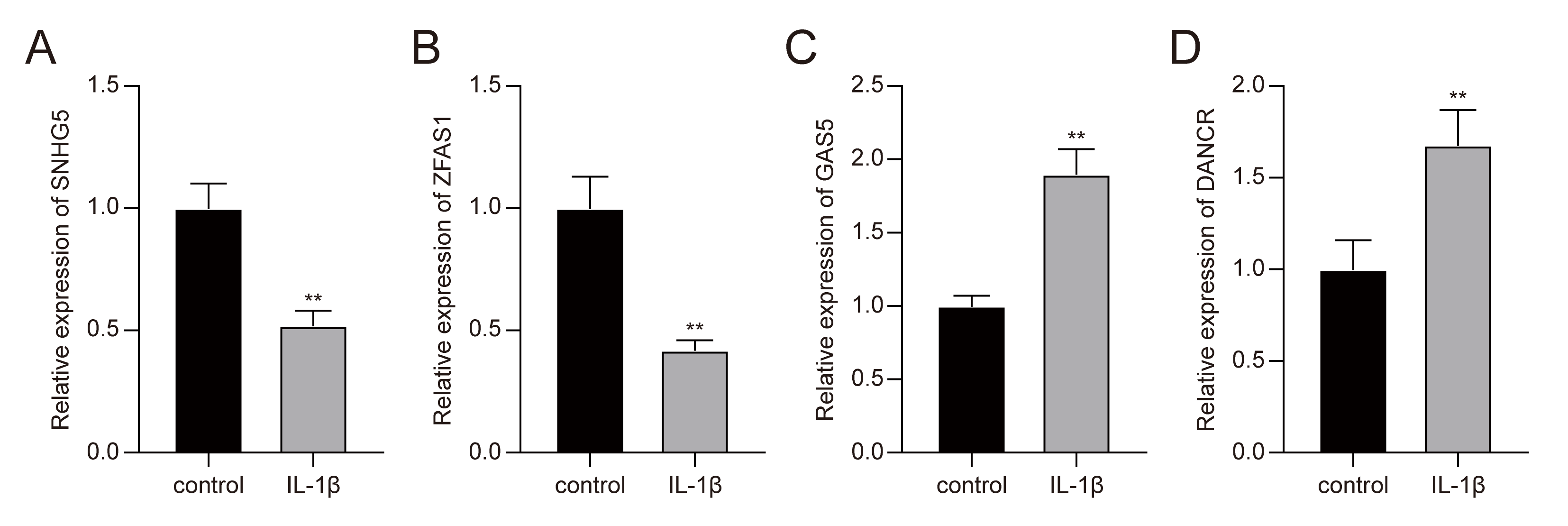

Supplement: Supplementary file 2 — Additional file 2: Figure S1. lncRNAs expression changes in chondrocytes treated with IL-1β. A–D The expression levels of four lncRNAs were determined by qRT-PCR analysis. The chondrocytes were treated with 10 ng/mL IL-1β for 24 h. **P < 0.01, compared with the control group [file 13018_2023_3802_MOESM2_ESM.tif]
