# Supplementary material for: LncRNA ZFAS1 protects chondrocytes from IL-1β-induced apoptosis and extracellular matrix degradation via regulating miR-7-5p/FLRT2 axis
Source: J Orthop Surg Res. 2023 Apr 25;18:320. doi: 10.1186/s13018-023-03802-9 (PMC10131303; doi:10.1186/s13018-023-03802-9)
Supplement: Supplementary file 3 — Additional file 3: Table S2. The list of the down-regulated genes in GSE110606 [file 13018_2023_3802_MOESM3_ESM.docx]

Table S2 The list of the down-regulated genes in GSE110606

| Gene Name | Ratio (OA/normal) | *P*-Value | Adjusted P-Value |
| --- | --- | --- | --- |
| MAGED2 | 0.50 | 0.04315 | 0.0459 |
| ASS1 | 0.50 | 0.0117 | 0.0182 |
| PITPNM3 | 0.50 | 0.01025 | 0.0166 |
| FZD1 | 0.49 | 0.00725 | 0.0130 |
| WWP2 | 0.49 | 0.0275 | 0.0335 |
| SLC29A1 | 0.49 | 0.01165 | 0.0182 |
| SLC5A3 | 0.49 | 0.00765 | 0.0136 |
| SLC39A14 | 0.48 | 0.0043 | 0.0090 |
| SSX2IP | 0.48 | 0.0282 | 0.0341 |
| LUM | 0.48 | 0.0362 | 0.0404 |
| C2orf40 | 0.48 | 0.0326 | 0.0383 |
| RCAN1 | 0.48 | 0.00855 | 0.0145 |
| TIMP3 | 0.47 | 0.00475 | 0.0096 |
| ASNS | 0.47 | 0.0061 | 0.0116 |
| PID1 | 0.47 | 0.01005 | 0.0165 |
| TNFRSF12A | 0.46 | 0.00305 | 0.0069 |
| TUBB2A | 0.46 | 0.0015 | 0.0042 |
| SPRY4 | 0.46 | 0.0466 | 0.0480 |
| SGCA | 0.46 | 0.01885 | 0.0256 |
| PSAT1 | 0.46 | 0.0016 | 0.0044 |
| CACHD1 | 0.46 | 0.00285 | 0.0065 |
| C9orf3 | 0.46 | 0.01015 | 0.0166 |
| PTER | 0.46 | 0.0178 | 0.0247 |
| SPDYE16 | 0.45 | 0.0353 | 0.0402 |
| ULBP3 | 0.45 | 0.0434 | 0.0461 |
| PTGFRN | 0.45 | 0.01905 | 0.0257 |
| BLOC1S2 | 0.45 | 0.041 | 0.0444 |
| FOXQ1 | 0.45 | 0.0035 | 0.0076 |
| TIPARP | 0.44 | 0.0022 | 0.0055 |
| UAP1 | 0.44 | 0.0014 | 0.0041 |
| HAPLN1 | 0.44 | 0.04805 | 0.0486 |
| LOXL3 | 0.43 | 0.044 | 0.0463 |
| FKBP5 | 0.43 | 0.0123 | 0.0189 |
| SMN2 | 0.43 | 0.0477 | 0.0486 |
| RSPO3 | 0.43 | 0.0297 | 0.0352 |
| CADM1 | 0.43 | 0.0188 | 0.0256 |
| ANKRD65 | 0.43 | 0.048 | 0.0486 |
| CYTL1 | 0.42 | 0.00265 | 0.0062 |
| BUB1 | 0.42 | 0.04825 | 0.0487 |
| SHROOM3 | 0.42 | 0.0055 | 0.0106 |
| GPR68 | 0.42 | 0.0086 | 0.0146 |
| PRIMA1 | 0.42 | 0.0411 | 0.0444 |
| FXYD6 | 0.42 | 0.04975 | 0.0498 |
| TIMP4 | 0.42 | 0.01785 | 0.0247 |
| PLPP1 | 0.42 | 0.0023 | 0.0057 |
| VLDLR | 0.42 | 0.0015 | 0.0042 |
| DMKN | 0.41 | 0.0113 | 0.0178 |
| SOX9 | 0.41 | 0.0006 | 0.0021 |
| DSP | 0.41 | 0.0013 | 0.0038 |
| DNER | 0.41 | 0.03515 | 0.0401 |
| NREP | 0.41 | 0.00485 | 0.0098 |
| KCNC4 | 0.41 | 0.0285 | 0.0343 |
| SIPA1L1 | 0.41 | 0.0125 | 0.0190 |
| CCDC36 | 0.41 | 0.0355 | 0.0402 |
| PNP | 0.41 | 0.0073 | 0.0130 |
| PRG4 | 0.40 | 0.0333 | 0.0386 |
| BMP6 | 0.40 | 0.0131 | 0.0196 |
| TNC | 0.40 | 0.04405 | 0.0463 |
| SH2D4A | 0.39 | 0.01825 | 0.0251 |
| SYBU | 0.39 | 0.0208 | 0.0277 |
| TNFAIP6 | 0.39 | 0.00055 | 0.0020 |
| ACOT11 | 0.39 | 0.0269 | 0.0331 |
| TBX4 | 0.39 | 0.01855 | 0.0253 |
| ICAM2 | 0.39 | 0.0486 | 0.0488 |
| RAI2 | 0.38 | 0.00975 | 0.0162 |
| F3 | 0.38 | 0.0003 | 0.0013 |
| MRAP2 | 0.37 | 0.00045 | 0.0018 |
| PODXL | 0.37 | 0.00175 | 0.0047 |
| CRTAC1 | 0.37 | 0.04265 | 0.0456 |
| GYPE | 0.37 | 0.04505 | 0.0468 |
| KIAA1644 | 0.37 | 0.00015 | 0.0008 |
| SLC2A9 | 0.37 | 0.04675 | 0.0480 |
| PKP1 | 0.36 | 0.00235 | 0.0057 |
| PTGS2 | 0.36 | 0.00105 | 0.0034 |
| GGH | 0.36 | 0.02195 | 0.0288 |
| STK26 | 0.36 | 0.00155 | 0.0043 |
| VWA5A | 0.36 | 0.00005 | 0.0003 |
| PDGFC | 0.36 | 0.00205 | 0.0053 |
| MFAP3L | 0.36 | 0.01835 | 0.0251 |
| KBTBD8 | 0.36 | 0.0402 | 0.0436 |
| SFN | 0.36 | 0.04505 | 0.0468 |
| SEMA3C | 0.36 | 0.0076 | 0.0135 |
| P3H2 | 0.36 | 0.008 | 0.0138 |
| LDLRAD4 | 0.35 | 0.02855 | 0.0343 |
| GPC6 | 0.35 | 0.00005 | 0.0003 |
| EPHX2 | 0.35 | 0.02325 | 0.0298 |
| RGS14 | 0.35 | 0.0435 | 0.0461 |
| ESPNL | 0.35 | 0.0347 | 0.0398 |
| EBF3 | 0.34 | 0.00545 | 0.0106 |
| HYAL1 | 0.34 | 0.00365 | 0.0079 |
| ELMO1 | 0.34 | 0.04405 | 0.0463 |
| COLEC12 | 0.34 | 0.00025 | 0.0011 |
| PTPRD | 0.34 | 0.04575 | 0.0473 |
| CST6 | 0.34 | 0.00815 | 0.0140 |
| ZFHX4 | 0.34 | 0.00085 | 0.0029 |
| NKAPL | 0.34 | 0.02165 | 0.0287 |
| COL11A1 | 0.33 | 0.00055 | 0.0020 |
| CDC45 | 0.33 | 0.0242 | 0.0306 |
| KCNQ3 | 0.33 | 0.00625 | 0.0118 |
| SLC35F2 | 0.33 | 0.0157 | 0.0227 |
| SCUBE1 | 0.33 | 0.04225 | 0.0454 |
| PLXDC2 | 0.33 | 0.001 | 0.0033 |
| SEC14L6 | 0.33 | 0.0244 | 0.0307 |
| GAS7 | 0.33 | 0.0233 | 0.0298 |
| EREG | 0.32 | 0.00255 | 0.0060 |
| DGKI | 0.32 | 0.02455 | 0.0308 |
| GPX3 | 0.32 | 0.00005 | 0.0003 |
| CCDC158 | 0.32 | 0.01145 | 0.0180 |
| FAM19A5 | 0.32 | 0.0013 | 0.0038 |
| SEMA3A | 0.31 | 0.0066 | 0.0122 |
| RGS22 | 0.31 | 0.04595 | 0.0474 |
| RGS16 | 0.31 | 0.0002 | 0.0010 |
| SLPI | 0.31 | 0.02725 | 0.0333 |
| NPTX1 | 0.30 | 0.04445 | 0.0466 |
| BEX2 | 0.30 | 0.0332 | 0.0386 |
| MYPN | 0.30 | 0.00055 | 0.0020 |
| AR | 0.30 | 0.0082 | 0.0140 |
| PDPN | 0.30 | 0.00005 | 0.0003 |
| DPP4 | 0.30 | 0.00055 | 0.0020 |
| CD4 | 0.30 | 0.00025 | 0.0011 |
| ULBP1 | 0.30 | 0.00105 | 0.0034 |
| WBSCR27 | 0.30 | 0.0089 | 0.0150 |
| PTPN6 | 0.29 | 0.01375 | 0.0204 |
| ADGRG2 | 0.29 | 0.00235 | 0.0057 |
| BMP2 | 0.29 | 0.0015 | 0.0042 |
| MILR1 | 0.29 | 0.0445 | 0.0466 |
| QPCT | 0.29 | 0.01255 | 0.0190 |
| MAOB | 0.29 | 0.004 | 0.0085 |
| DPF1 | 0.29 | 0.01985 | 0.0266 |
| PCOLCE2 | 0.29 | 0.00005 | 0.0003 |
| CKB | 0.28 | 0.00065 | 0.0023 |
| SEMA5A | 0.28 | 0.00455 | 0.0092 |
| NFATC2 | 0.28 | 0.00055 | 0.0020 |
| CD24 | 0.28 | 0.0265 | 0.0328 |
| IL3RA | 0.28 | 0.026 | 0.0322 |
| SCIN | 0.28 | 0.0348 | 0.0399 |
| SERPINI1 | 0.28 | 0.0055 | 0.0106 |
| CHAC1 | 0.28 | 0.00005 | 0.0003 |
| LMX1B | 0.28 | 0.002 | 0.0052 |
| FAM134B | 0.28 | 0.008 | 0.0138 |
| APBB1IP | 0.28 | 0.0008 | 0.0027 |
| APOD | 0.28 | 0.0033 | 0.0074 |
| IQCA1 | 0.28 | 0.0109 | 0.0174 |
| ATF5 | 0.27 | 0.00125 | 0.0038 |
| ROBO3 | 0.27 | 0.0077 | 0.0136 |
| KIAA1324L | 0.27 | 0.0223 | 0.0290 |
| CLMN | 0.27 | 0.031 | 0.0366 |
| ADAMTSL3 | 0.27 | 0.0004 | 0.0016 |
| NOMO3 | 0.27 | 0.024 | 0.0304 |
| FLRT2 | 0.27 | 0.0033 | 0.0074 |
| TNNC1 | 0.26 | 0.0108 | 0.0173 |
| CLIC6 | 0.26 | 0.00105 | 0.0034 |
| ACTC1 | 0.26 | 0.03805 | 0.0418 |
| FNDC1 | 0.26 | 0.00005 | 0.0003 |
| RAMP1 | 0.26 | 0.00235 | 0.0057 |
| KCNB1 | 0.26 | 0.0038 | 0.0081 |
| KRTAP2-3 | 0.26 | 0.03035 | 0.0359 |
| LY6K | 0.26 | 0.0049 | 0.0098 |
| IL13RA2 | 0.24 | 0.00005 | 0.0003 |
| HRCT1 | 0.24 | 0.0021 | 0.0053 |
| ESM1 | 0.24 | 0.0011 | 0.0035 |
| ADCY2 | 0.24 | 0.01295 | 0.0194 |
| AKR1E2 | 0.24 | 0.01205 | 0.0187 |
| FAM86B1 | 0.24 | 0.00015 | 0.0008 |
| LGR5 | 0.24 | 0.00275 | 0.0064 |
| MKX | 0.24 | 0.00055 | 0.0020 |
| BST2 | 0.23 | 0.01515 | 0.0220 |
| FGF1 | 0.23 | 0.0023 | 0.0057 |
| ITIH5 | 0.23 | 0.00005 | 0.0003 |
| TNFRSF1B | 0.23 | 0.0037 | 0.0080 |
| RASGEF1A | 0.22 | 0.02955 | 0.0351 |
| GPAT2 | 0.22 | 0.01495 | 0.0218 |
| MECOM | 0.22 | 0.01725 | 0.0242 |
| KCNQ5 | 0.22 | 0.00005 | 0.0003 |
| ANK3 | 0.22 | 0.0162 | 0.0233 |
| SCRG1 | 0.22 | 0.00005 | 0.0003 |
| SERPINE2 | 0.22 | 0.00005 | 0.0003 |
| LBH | 0.21 | 0.00005 | 0.0003 |
| DSG2 | 0.21 | 0.00005 | 0.0003 |
| FGF13 | 0.20 | 0.0145 | 0.0213 |
| B4GALNT3 | 0.20 | 0.00285 | 0.0065 |
| FGFR3 | 0.20 | 0.0002 | 0.0010 |
| FAT3 | 0.20 | 0.0458 | 0.0473 |
| EN1 | 0.20 | 0.0011 | 0.0035 |
| COL15A1 | 0.20 | 0.00005 | 0.0003 |
| ANOS1 | 0.19 | 0.00005 | 0.0003 |
| KCNK2 | 0.19 | 0.00025 | 0.0011 |
| ENPP1 | 0.19 | 0.00005 | 0.0003 |
| DUSP15 | 0.17 | 0.04695 | 0.0480 |
| ZNF385D | 0.17 | 0.00125 | 0.0038 |
| FGFBP2 | 0.17 | 0.0121 | 0.0187 |
| SERPINA1 | 0.17 | 0.00025 | 0.0011 |
| NEBL | 0.17 | 0.00005 | 0.0003 |
| SPX | 0.16 | 0.00015 | 0.0008 |
| C5orf38 | 0.16 | 0.008 | 0.0138 |
| ICA1 | 0.16 | 0.00335 | 0.0075 |
| FBXO2 | 0.16 | 0.0065 | 0.0122 |
| CBSL | 0.15 | 0.00425 | 0.0090 |
| FBXO27 | 0.15 | 0.0165 | 0.0236 |
| AOC2 | 0.14 | 0.00005 | 0.0003 |
| MCOLN2 | 0.14 | 0.00195 | 0.0051 |
| ISM1 | 0.14 | 0.00005 | 0.0003 |
| ALDH3A1 | 0.14 | 0.00005 | 0.0003 |
| AL354822.1 | 0.14 | 0.00015 | 0.0008 |
| AGR2 | 0.13 | 0.0126 | 0.0190 |
| ZNF385B | 0.13 | 0.0006 | 0.0021 |
| MDFI | 0.13 | 0.0002 | 0.0010 |
| SDK2 | 0.13 | 0.00005 | 0.0003 |
| SPATC1L | 0.12 | 0.0001 | 0.0006 |
| PEG3 | 0.12 | 0.04355 | 0.0461 |
| GLDN | 0.12 | 0.00005 | 0.0003 |
| SERPINA5 | 0.12 | 0.03515 | 0.0401 |
| CCDC85A | 0.11 | 0.00005 | 0.0003 |
| SERPINA3 | 0.11 | 0.0011 | 0.0035 |
| STXBP2 | 0.11 | 0.00455 | 0.0092 |
| SERPINA3 | 0.10 | 0.00005 | 0.0003 |
| CHRDL1 | 0.10 | 0.00005 | 0.0003 |
| FRZB | 0.09 | 0.00005 | 0.0003 |
| ADRA2C | 0.09 | 0.00005 | 0.0003 |
| C2orf82 | 0.09 | 0.03655 | 0.0406 |
| CHI3L2 | 0.09 | 0.00015 | 0.0008 |
| ATP1A3 | 0.08 | 0.0002 | 0.0010 |
| COL9A2 | 0.08 | 0.00005 | 0.0003 |
| AC145212.4 | 0.08 | 0.00005 | 0.0003 |
| RBP4 | 0.08 | 0.00005 | 0.0003 |
| COMP | 0.07 | 0.00005 | 0.0003 |
| S100A1 | 0.07 | 0.034 | 0.0393 |
| SHANK2 | 0.06 | 0.00005 | 0.0003 |
| S100B | 0.06 | 0.00005 | 0.0003 |
| CA2 | 0.05 | 0.0021 | 0.0053 |
| COL9A3 | 0.04 | 0.00005 | 0.0003 |
| COL11A2 | 0.04 | 0.00005 | 0.0003 |
| PLA2G2A | 0.01 | 0.00005 | 0.0003 |
